# Quick response codes in the breakfast cereal aisle: prevalence, landing page, and product characteristics

**DOI:** 10.1093/heapro/daag044

**Published:** 2026-04-06

**Authors:** Laura Bathie, Maria Camille Louise Chen, Amy Leahy, Bella Sträuli, Simone Pettigrew, Eden M Barrett

**Affiliations:** The George Institute for Global Health, Faculty of Medicine & Health, University of New South Wales, PO Box 6366, Sydney, NSW 1466, Australia; The George Institute for Global Health, Faculty of Medicine & Health, University of New South Wales, PO Box 6366, Sydney, NSW 1466, Australia; School of Population Health, University of New South Wales, Building C29 HTH Level 5, Sydney, NSW 2052, Australia; The George Institute for Global Health, Faculty of Medicine & Health, University of New South Wales, PO Box 6366, Sydney, NSW 1466, Australia; School of Population Health, University of New South Wales, Building C29 HTH Level 5, Sydney, NSW 2052, Australia; The George Institute for Global Health, Faculty of Medicine & Health, University of New South Wales, PO Box 6366, Sydney, NSW 1466, Australia; School of Medicine and Dentistry, Gold Coast Griffith University, Parklands Drive, Southport, QLD 4222, Australia; The George Institute for Global Health, Faculty of Medicine & Health, University of New South Wales, PO Box 6366, Sydney, NSW 1466, Australia; The George Institute for Global Health, Faculty of Medicine & Health, University of New South Wales, PO Box 6366, Sydney, NSW 1466, Australia

**Keywords:** QR code, digital label, food label, packaged food, nutrition information

## Abstract

Digital labelling technologies, including quick response (QR) codes, are increasingly used in the global packaged food supply to provide additional product information. While regulation in most jurisdictions requires essential nutrition and ingredient information to be displayed directly on the physical label, concerns exist among researchers and consumer groups that QR codes may come to replace these key details, limiting access to essential information required for informed decision-making. This study aimed to assess the prevalence of QR codes on breakfast cereals in Australia, the types of information the codes provide and the healthfulness (measured by Health Star Rating and Nova systems) of products that feature them. In a sample of 483 breakfast cereals, 16% displayed QR codes, most frequently located on the back of the pack (72%). There were no significant differences between products with or without QR codes in median Health Star Rating (4.0 vs. 4.0; *P* = .832) or the proportion of ultra-processed products (83% vs. 76%; *P* = .136). QR codes were primarily linked to websites promoting the product (e.g. 80% featured product recipes), with none providing nutrition information besides claims. Our study provides evidence that, although QR codes are not yet widespread on breakfast cereals in Australia, they are currently being used mostly as additional marketing space rather than to provide key nutrition information to consumers. Their prevalence may increase as digital labelling technologies gain momentum globally. Regulatory oversight that prioritizes access to key nutrition information is important to ensure consumers can easily access the information required to make informed decisions.

Contribution to Health Promotion

- QR code prevalence on food products and the nature of the information they provide are largely unknown.
- QR codes featured on 16% of our breakfast cereal sample, mainly on the back of packs.
- There was no pattern in the presence of QR codes based on product healthfulness.
- Most of the linked websites contained marketing material rather than key nutrition information.
- Our results highlight the need for monitoring and regulation to ensure consumers can access the information needed for informed decisions.

## Introduction

Quick response (QR) codes—a type of digital labelling technology—are becoming increasingly prominent in the global food sector (Li et al. 2024). In the packaged food supply, this trend is transforming how consumers access product information (Li and Messer 2019). Instead of only accessing product information via the physical label, QR codes can enable digital access to a broader range of details via a scannable two-dimensional square printed on a product’s package (Li et al. 2024). This includes entirely new types of information such as real-time product information (e.g. when paired with smart sensor technology) (Chrysochou and Tiganis 2025) and additional content that would not have otherwise fit on a traditional label (Li et al. 2024, Bonioli and Bazzani 2025). Additional content may include marketing material like videos or articles that strengthen brand image or provide entertainment, recipes that inspire cooking and rewards such as discount codes (Rotsios et al. 2022, Li et al. 2024). QR codes are thus highly beneficial for manufacturers, as they can support marketing efforts and brand engagement to enhance product appeal (Huda et al. 2021, Li et al. 2024, Kokole et al. 2025). Given these advantages, there is increasing industry interest globally in digitalizing product information (BEUC 2021, European Commission 2022, Australian Food and Grocery Council 2025). This includes using QR codes to replace key details traditionally printed directly on the label (e.g. nutrition and ingredient information) (BEUC 2021, Werle et al. 2022, Gaudeul and Krawczyk 2023), for which there is some regulatory support (with respect to wine labelling) (Directorate-General for Agriculture and Rural Development 2023). This is concerning because reliance on QR codes alone to provide product information could limit consumers’ immediate access to essential information required to make informed choices (European Commission 2022, Werle et al. 2022, Gaudeul and Krawczyk 2023, Food Standards Australia New Zealand 2024).

Experimental evidence reinforces these concerns, as it suggests that consumers are often unwilling to access information via QR codes (Li and Messer 2019, Werle et al. 2022, Gaudeul and Krawczyk 2023), favouring information that is immediately visually available (Werle et al. 2022). A review found that when unprompted, consumers scanned QR codes across a range of food product categories less than a quarter of the time (Bonioli and Bazzani 2025). Perceived ease of use was identified as a critical blockage to engagement, with additional effort, technological barriers, and time constraints identified as key hurdles. While most jurisdictions still require nutrition and ingredient information to be printed on physical labels (European Parliament and Council of the European Union 2011, Food Standards Australia New Zealand 2016, Afroza et al. 2024), growing interest in digitalization in the food industry has prompted researchers and consumer groups to caution against movement towards relying solely on digital technologies to provide food information (BEUC 2021, Werle et al. 2022).

Despite manufacturer support for QR codes, research on the prevalence of these codes in the food supply is sparse, with most of the limited work to date focussing on consumer understanding and use of QR codes (Li and Messer 2019, Werle et al. 2022, Gaudeul and Krawczyk 2023). Some work in the context of alcohol has found highly variable prevalence of QR codes between studies (4%–31%) (Sarasa-Renedo et al. 2022, Padilla-Cruz et al. 2024, Kokole et al. 2025). The most recent of these studies also analysed the content of the linked websites, finding brand information to be most common (Kokole et al. 2025). Research exploring QR code use on food products is timely given the increased interest in digital transformation initiatives in the food industry globally (Gordon 2024).

The present study aimed to address the paucity of evidence regarding QR code use on food products. Specifically, this study aimed to assess the prevalence of QR codes on breakfast cereal product labels in the Australian packaged food supply in 2024, analyse the linked content, and examine whether products with QR codes differ in healthfulness compared to those without. The category of breakfast cereals was selected as breakfast cereals are widely consumed in Australia—approximately one third of the population reported consuming breakfast cereals in the 24-hour period prior in the most recent National Nutrition and Physical Activity Survey (Australian Bureau of Statistics 2023)—indicating greater potential impact on diet-related health outcomes relative to other food categories. Breakfast cereals also vary significantly in nutritional quality (e.g. sugar, fibre, and whole grain content) and degree of processing (e.g. rolled oats vs. ready-to-eat cereals) (Croisier et al. 2021), thus exemplifying their potential degree of impact on health. This makes them an informative case study for examining QR codes among commercial foods.

Identifying the types of information provided by linked content is especially important to determine whether these digital tools can facilitate more informed and health-conscious food choices. Certain forms of misleading advertising appear to be increasingly prevalent in the digital marketplace (Whalen et al. 2018, European Commission 2021, Ares et al. 2023, Australian Competition and Consumer Commission 2023a)— namely, product ‘washing’ such as ‘healthwashing’ (i.e. ‘when health is used as a selling argument by implying benefits or unharmful effects of a product when the reality is different’; Delerm et al. 2023) and ‘greenwashing’ (i.e. ‘Where claims misrepresent the environmental impact associated with a business or the goods and services it supplies’; Australian Competition and Consumer Commission 2023b). This development underscores the need for ongoing monitoring of this content. Further, it is important to identify whether there are any differences in healthfulness—a concept encompassing the distinct but complementary dimensions of nutritional quality and level of processing (Barrett et al. 2023)—between products with and without QR codes. This approach permits assessment of whether this digital labelling technology clusters on specific product types (e.g. low nutritional quality or ultra-processed products)—an important consideration given live policy discussions around digital labelling (BEUC 2021, Food Standards Australia New Zealand 2024) and the potential for QR codes to enhance brand engagement and product appeal (Huda et al. 2021, Li et al. 2024, Kokole et al. 2025). Benchmarking current practice will enable monitoring of whether QR codes are being used primarily as a promotional tool for less healthy, ultra-processed products, helping to inform future regulatory oversight.

## Materials and methods

We used data from The George Institute for Global Health’s 2024 Australian FoodSwitch Monitored Dataset (FoodSwitch). FoodSwitch contains labelling and nutritional composition data for packaged foods available for sale in major supermarkets across Sydney, Australia (Coyle et al. 2022). These data have been annually collected since 2013. A previous analysis indicated that annual FoodSwitch collections are representative of approximately 97% of packaged food products purchased in Australia (Coyle et al. 2022). We also used the FoodSwitch Content Management System (CMS), which contains photographs of all sides of all products within FoodSwitch.

Products in FoodSwitch are categorized using the Global Food Monitoring Group’s original hierarchical system into major categories (such as cereal and grain products), minor categories (such as breakfast cereals), and subcategories (such as hot cereal) (Neal et al. 2013). In this study, all products within the minor food category of ‘Breakfast cereals’ (*n* = 483) were included.

Products were rated according to Australia’s voluntary front-of-pack nutrition labelling system, the Health Star Rating (HSR), which rates products from 0.5 to 5 stars based on nutritional quality. FoodSwitch contains HSR scores that were manually calculated according to the official underlying nutrient profiling algorithm, as previously described (Barrett et al. 2025). The calculation involved determining a product’s total ‘negative’ points based on per 100-g values of energy, saturated fat, total sugar, and sodium and modifying the final score based on ‘positive’ points for a product’s percentage of fruit, vegetable, nut and legume, protein, and fibre content (also per 100 g). For consistency, we used the FoodSwitch-calculated HSR score for all products, regardless of whether the product packaging reported a HSR.

Ultra-processed food (UPF) is associated with adverse health outcomes (Machado et al. 2025) and now comprises a significant proportion of Australian diets (Coyle et al. 2022). Therefore, the Nova classification system—which categorizes foods according to their degree of processing—was used to categorize breakfast cereals in our sample as either UPF (Nova group 4) or non-UPF (Nova groups 1–3) (Monteiro et al. 2018). UPFs were identified by the presence of a cosmetic additive (e.g. flavours and colours) or industrial food substance (e.g. hydrogenated oils and high-fructose corn syrup) in the product’s ingredients list (Monteiro et al. 2019, Barrett et al. 2023). Photographs of products in the CMS were used to determine the presence and location of QR codes on breakfast cereals, as well as the content of the linked websites.

Among products displaying QR codes, we recorded the location of the QR code (e.g. back or side of the package) and the types of information provided on the associated landing pages. The QR code was scanned with a smartphone (using the product images available in the CMS) and the contents of the entire landing page were analysed. The information found was categorized using deductive (‘Recipes’, ‘References to sustainability’, ‘Recycling information’, ‘Nutrition information’, and ‘Health claims’; Kokole et al. 2025) and inductive (‘Country-of-origin information’, ‘Nutrition claims’, ‘Links to purchase the product online’, and ‘Discounts for bulk purchasing’) codes. In the present study, these codes were defined as follows: (i) product recipes referred to procedures for cooking meals and snacks that incorporated the breakfast cereal; (ii) references to sustainability included sustainability claims, which were any claims relating to the environment or sustainability and more detailed information relating to production methods; (iii) recycling information included any reference to the recyclability of the packaging; (iv) nutrition information referred to a nutrition information panel that provided the per 100 g/ml levels of essential nutrients (protein, fat, saturated fat, carbohydrates, sugar, and sodium); (v) health claims were statements that related a nutrient to health; (vi) country-of-origin information referred to any information pertaining to where the product was made and included the regulated standard mark (Australian Government 2016) or unregulated statements (e.g. ‘Aussie-made’ or ‘Proudly Australian’); (vii) nutrition claims were statements about nutrients or ingredients (e.g. ‘rich in vitamins and minerals’ or ‘vegan friendly’), as well as vague claims about the quality of the ingredients (e.g. ‘wholesome ingredients’); (viii) links to purchase products were links embedded in the landing page that allowed the user to purchase the company’s products online; and (ix) discounts for bulk purchasing were discounts applied to online purchases that exceeded a specified minimum spend (e.g. ‘Free shipping for orders over $70 Australia-wide’). Content assessment was made independently by two authors (M.C.L.C., A.L.) and a third author (E.M.B.) resolved any disagreements. Disagreement between the coders was very low (<5% for any one variable and overall), likely due to the use of predetermined codes, as previously described.

We calculated the frequency and proportion of breakfast cereals with and without QR codes. We compared the median HSR and interquartile range (IQR) between these groups using the Mann–Whitney U test. Proportions of products classified as UPF were compared using a two-proportion z-test. We followed previous methods to combine Nova groups 1–3 (non-UPF) and compare it to Nova group 4 (UPF) (Barrett et al. 2023). This approach is supported by literature which typically focuses on contrasting UPFs with all other food groups due to the negative implications of diets high in UPF (Elizabeth et al. 2020). Analyses were conducted in Stata version 18.0. This study did not use human or animal data and therefore ethics approval was not required.

## Results

In total, 16% (*n* = 78/483) of the assessed breakfast cereals featured QR codes, which were primarily located on the back of packs (72%, *n* = 56/78). There was no difference in the median HSR between products displaying and not displaying a QR code [4.0 (IQR 4.0, 4.5) vs. 4.0 (IQR 4.0, 4.5), *P* = .832]. Although the difference was not significant, a greater proportion of products displaying a QR code were UPF compared to those without a QR code (83%, *n* = 65/78 vs. 76%, *n* = 306/405, *P* = .136).

All the QR codes (100%, *n* = 78/78) led to a brand/product website, most of which contained promotional content. For instance, every linked website (100%, *n* = 78/78) featured country-of-origin information and the vast majority referenced Australia (e.g. ‘A family owned Australian Business’ and ‘Aussie-made’) (Table 1). Product recipes were present on 80% (*n* = 62/78) of linked websites (e.g. ‘Christmas Rocky Road with Maple Clusters’). Health or nutrition claims were also heavily featured on linked websites (71%*, n*  *=* 55/78). Most health and nutrition claims flagged the presence of ingredients or nutrients (e.g. whole grains and fibre) or the reduction or absence of specific nutrients (e.g. ‘low sugar’ and ‘gluten free’), while some referred to the nature of the ingredients generally (e.g. ‘wholesome ingredients’). Many products had a combination of both types of claims. These claims were either present on the linked website or on product photos embedded in the linked website.

**Table 1:** Information provided via QR codes on breakfast cereals (*n* = 78 products).

Information type	Information present on QR code landing page	
	*n*	%
Country-of-origin information	78	100
Product recipes	62	80
Health or nutrition claims^a^	55	71
Sustainable production information^b^	42	54
Sustainability claims^c^	40	51
Link to purchase products	26	33
Recycling information	17	22
Discount for bulk purchasing	15	19
Nutrition information panel	0	0

^a^Any claims relating to health (e.g. ‘contains beta glucan of oats and prebiotic inulin for good gut health’), nutrients (e.g. ‘low sugar’), or ingredients (e.g. ‘wholesome ingredients’). ^b^Any more detailed information regarding sustainable or environmentally friendly production methods, e.g. information regarding renewable energy sources used to power a company’s bakehouse. ^c^Any claims relating to the environment or sustainability, e.g. ‘carbon neutral’ and ‘improves soil health’.

Over half of the linked websites (54%, *n* = 42/78) included information regarding sustainable or environmentally friendly production methods (e.g. one company provided information on their ‘Cool Soil Initiative’). Finally, sustainability claims were another type of promotional content present on most of the linked websites (51%, *n* = 40/78). These claims were typically short statements asserting environmental benefits or sustainability outcomes (e.g. ‘strong passion for sustainability’ or ‘plate2farm transparency’).

## Discussion

In our analysis of breakfast cereals available for sale in Australia, about one in six products (16%) displayed a QR code, mostly located on the back of packs. There was no clear pattern in QR code presence based on product healthfulness. All the QR codes linked to a brand/product website and the most commonly featured content included country-of-origin information, product recipes and health, nutrition and references to sustainability.

The QR code prevalence found in the present study was about half that observed in a study analysing alcohol products in European countries in 2024 (Kokole et al. 2025). One possible reason for this is the introduction of Regulation (EU) 2021/2117, which applies to wines produced from December 2023 and allows mandatory nutritional and ingredient information to be provided electronically (European Parliament and Council of the European Union 2021, Directorate-General for Agriculture and Rural Development 2023). Similar to the findings of the alcohol study (Kokole et al. 2025), the majority of QR codes were situated on the back of breakfast cereals, making them less accessible to consumers. This lends credence to concerns about consumers’ ease of access to the information provided via QR codes, particularly if the information is essential to make informed decisions (e.g. nutrition information).

Our study provides evidence that QR codes are not yet widespread on breakfast cereals in Australia and the linked content primarily consists of marketing material rather than key nutrition information. This reflects the current regulatory environment in Australia, where the Food Standards Code does not require the inclusion of specific information via QR codes but does require key nutrition details—such as the nutrition information panel and ingredients list—to be printed directly on the physical label (Food Standards Australia New Zealand 2016). However, with growing interest in expanding the use of digital labels (BEUC 2021, European Commission 2022, Australian Food and Grocery Council 2025), regulatory oversight must ensure that key nutrition information remains readily accessible on the physical label. In a rapidly evolving digital environment—where regulatory uncertainty could be exploited (McKerchar et al. 2023)—clear, enforceable rules are important to uphold consumers’ fundamental right to access the information required to make informed choices.

Our study demonstrated that much of the content linked via QR codes featured health or nutrition claims or sustainability messaging. While this content can provide useful information, it can be problematic if it is false, deceptive or misleading, including if the claims are too vague to substantiate (Heiss et al. 2021, Delerm et al. 2023, Australian Competition and Consumer Commission 2023b). Some claims in our sample could potentially fall into these categories. For example, the linked content for some low-HSR, ultra-processed products included claims such as ‘natural ingredients’ and ‘real food’. These ‘soft claims’ fall outside regulatory requirements, lack specific details and scientific validation and could mislead consumers to perceive products as healthier or less processed than they are (Arraztio-Cordoba et al. 2025). In addition, many products featured regulated nutrition claims such as ‘low sugar’ or ‘high protein’. Recent research suggests such claims increase perceived healthiness, choice and purchases of the products bearing the claims (Kelly et al. 2024). This is concerning because food products available for sale in Australia are not required to meet a minimum healthiness threshold to display a nutrition claim—unlike health claims, which are subject to stricter criteria (Food Standards Australia New Zealand 2025). Consequently, nutrition claims do not guarantee overall product healthiness and could mislead consumers (Hughes et al. 2013).

While product ‘washing’ is not a new phenomenon, the advent of digital communication has multiplied the number of channels through which this can occur (Delerm et al. 2023), thus increasing the challenge of regulatory enforcement. Frequent exposure to such messaging could ‘prime’ consumers to associate ‘washed’ products with the health or environmental context in which they are presented, potentially increasing the likelihood of them choosing those products (Heiss et al. 2021). To mitigate this risk, regulatory oversight must ensure that information provided via digital labels is accurate, trustworthy and not misleading to protect consumers. Regulators should be equipped with sufficient resources to monitor compliance and enforce penalties that act as meaningful deterrents against breaches.

Our analyses contribute to the emerging body of evidence regarding the use of QR codes in the food supply, providing an important benchmark for monitoring future developments in this area. Strengths include the use of a comprehensive, nationally representative database, FoodSwitch. However, our study was limited to products available in the Australian packaged food supply in one food category and findings may vary across countries and food categories. Future research should explore the use of QR codes in other product categories and food environments globally. As digital labelling increases in prominence across food environments, this is an important area for future research.

## Conclusion

Our study found that around one in six breakfast cereals available in Australia in 2024 featured a QR code, typically on the back of the pack and mainly used to provide additional marketing material to consumers. As the trend towards digital labelling continues to gain momentum, regulatory oversight is warranted. Such oversight should prioritize access to key nutrition information and ensure the accuracy of digital information to protect consumer rights and prevent them from being misled.

## Data Availability

The data that support the findings of this study are available from FoodSwitch, but restrictions apply to the availability of these data, which were used under licence for the current study and so are not publicly available.
